# Portopulmonary Hypertension: From Bench to Bedside

**DOI:** 10.3389/fmed.2020.569413

**Published:** 2020-11-03

**Authors:** Christopher Thomas, Vladimir Glinskii, Vinicio de Jesus Perez, Sandeep Sahay

**Affiliations:** ^1^Division of Pulmonary, Allergy & Critical Care Medicine, Stanford University School of Medicine, Stanford, CA, United States; ^2^Houston Methodist Hospital Lung Center, Houston Methodist Hospital, Houston, TX, United States

**Keywords:** portopulmonary hypertension, pulmonary arterial hypertension, portal hypertension, liver transplant, MELD exception

## Abstract

Portopulmonary hypertension (PoPH) is defined as pulmonary arterial hypertension (PAH) associated with portal hypertension and is a subset of Group 1 pulmonary hypertension (PH). PoPH is a cause of significant morbidity and mortality in patients with portal hypertension with or without liver disease. Significant strides in elucidating the pathogenesis, effective screening algorithms, accurate diagnoses, and treatment options have been made in past 20 years. Survival of PoPH has remained poor compared to IPAH and other forms of PAH. Recently, the first randomized controlled trial was done in this patient population and showed promising results with PAH specific therapy. Despite positive effects on hemodynamics and functional outcomes, it is unclear whether PAH specific therapy has a beneficial effect on long term survival or transplant outcomes. In this review, we will discuss the epidemiology, pathophysiology, clinical and hemodynamic characteristics of PoPH. Additionally, this review will highlight the lacunae in our current management strategy, challenges faced and will provide direction to potentially useful futuristic management strategies.

## Introduction and Background

Pulmonary arterial hypertension (PAH) is a chronic progressive disease characterized by elevated pulmonary artery pressure and pulmonary vascular resistance (PVR) eventually leading to right heart failure and premature death. PAH associated with portal hypertension is called portopulmonary hypertension (PoPH), and is a subset of Group 1 PH (1). Similar to other causes of PAH, PoPH is characterized by the presence of intimal proliferation, medial hypertrophy, adventitial fibrosis of the muscular arteries and plexiform arteriopathy, and is thought to be histopathologically indistinguishable from other PAH phenotypes (2–4). Importantly, there does not seem to be a link between the presence or severity of PoPH with the degree of underlying liver dysfunction or hepatic venous pressure gradient (5, 6).

Patients with PoPH may be treated with PAH specific therapies, which may decrease the severity of the disease, improve functional parameters and hemodynamics, and allow for liver transplant (LT) (7). Despite a rapidly evolving understanding of PoPH, challenges remain in the diagnosis, treatment and transplantation of patients with PoPH. In this review, we will discuss the latest expert opinion in diagnosis and management of PoPH, as well as identify key areas of future research focus.

## Demographics and Clinical Characteristics of Patients With PoPH

The exact prevalence of PoPH is difficult to determine. The annual incidence of all types of PAH is <10 patients per million population, and PoPH patients are a subset of this group (8). PoPH was the third most common form of PAH in a population-based epidemiologic study in France (9). Historically, PoPH was thought to be 5–10% of all patients with PAH (10, 11). More recently, French authors have reported that the proportion of newly diagnosed (incident) patients with PoPH is as high as 15% of all patients with PAH, and is continuing to rise as wider screening practices are adopted (12). PoPH is thought to be present in anywhere between 2 and 10% of patients with portal hypertension (6, 13). Furthermore, while the vast majority of cases of PoPH are in patients with portal hypertension related to cirrhosis, non-cirrhotic causes of portal hypertension (including portal vein thrombosis, granulomatous disease, auto-immune disorders, drug reactions, infections, and congenital abnormalities) are also important contributors (14, 15). McDonnell et al. showed a prevalence of histopathologic changes of PAH of 0.61% in autopsies of patients with cirrhosis (16), and small cohort studies have shown prevalence of PoPH in patients with cirrhosis undergoing transplant evaluation to be between 5 and 6% (17–19).

**Figure 1:**
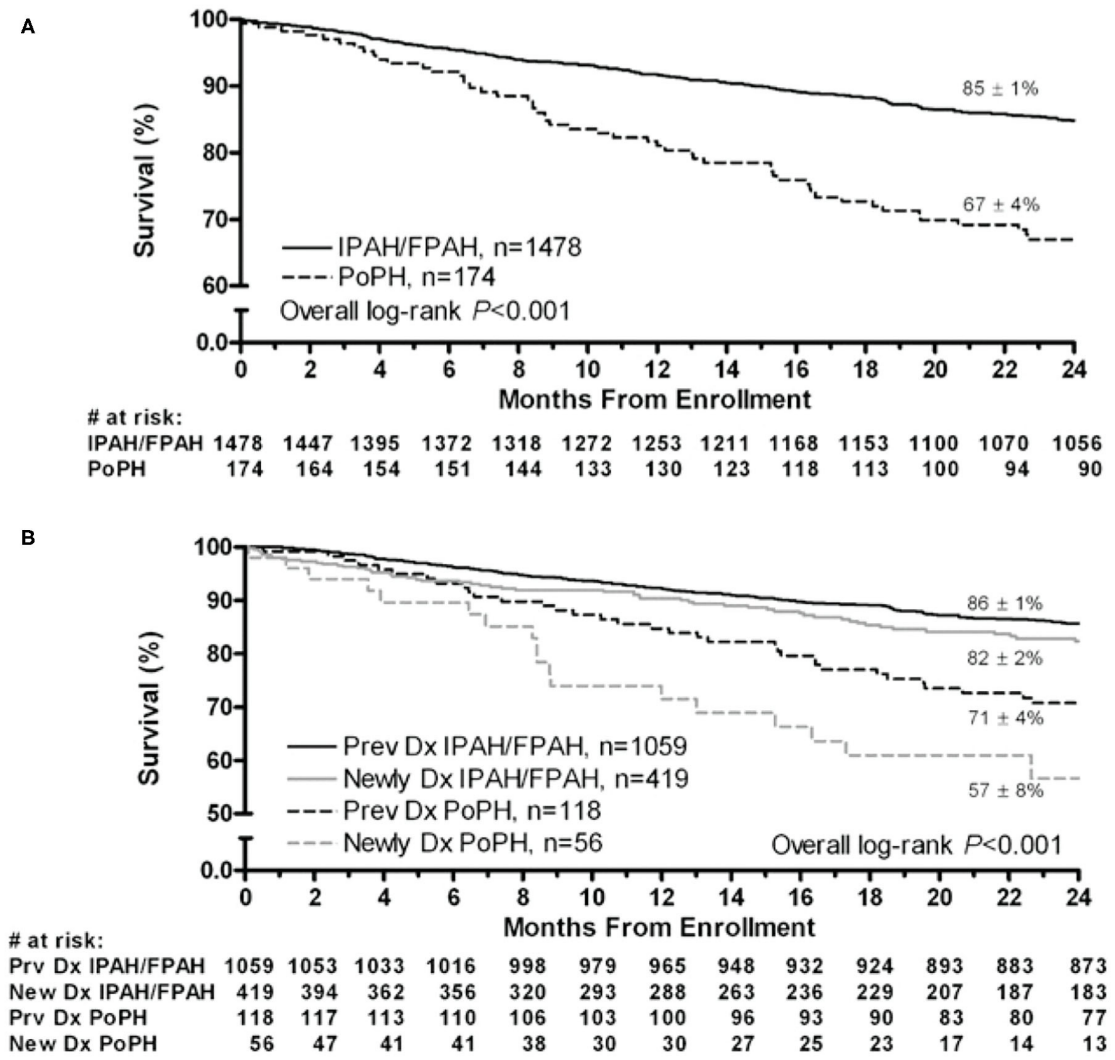
Two-year survival from enrollment. **(A)** Patients with PoPH and IPAH/FPAH. **(B)** Stratified by duration of disease from enrollment. Dx, diagnosis; FPAH, familial pulmonary arterial hypertension; IPAH, idiopathic pulmonary arterial hypertension; PoPH, portopulmonary hypertension. Reproduced with permission of Elsevier: Chest 141 (4) 906-915; doi: 10.1378/chest.11-0160 Epub 21 July 2011.

**Figure 2:**
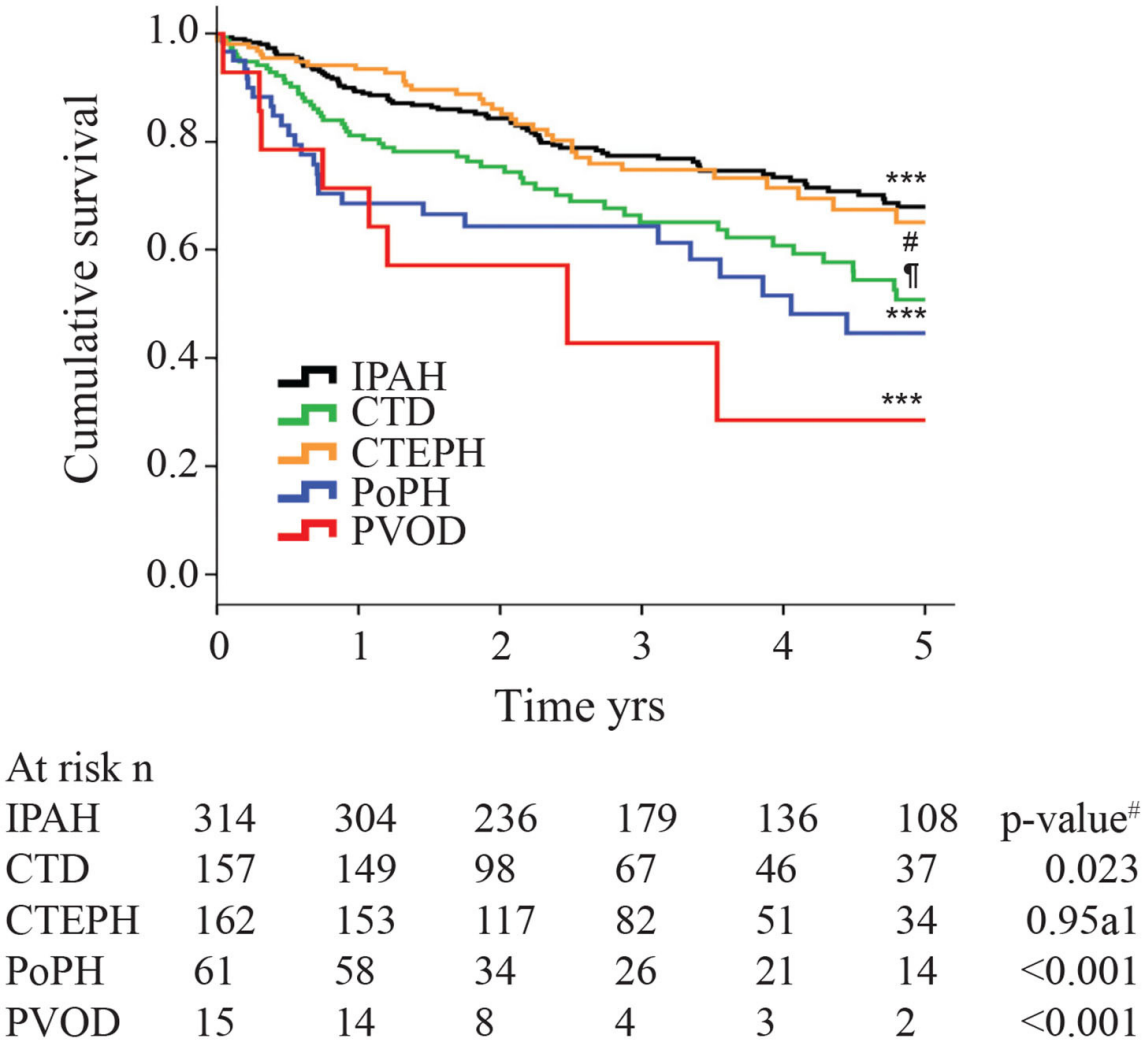
Kaplan-Meier estimates of 5-years survival from time of diagnosis in different pulmonary hypertension subtypes. IPAH, Idiopathic Pulmonary arterial hypertension; CTD, connective tissue disease; CTEPH, chronic thromboembolic pulmonary hypertension; PoPH, portopulmonary hypertension; PVOD, pulmonary veno-occlusive disease. The *p*-value for the overall comparison is <0.001. ^#^ compared with IPAH. Reproduced with permission of the © ERS 2020: European Respiratory Journal 40 (3) 596-603; doi: 10.1183/09031936.00101211 Published 31 August 2012.

Although the hemodynamic profile of patients with PoPH is better than in patients with idiopathic/familial PAH (IPAH/HPAH), their overall mortality is similar or worse (12, 20–22). The French Pulmonary Hypertension Registry (FHPR) shows a 1, 3, and 5-years survival of 84, 69, and 51%, respectively, for patients with PoPH (12), which is similar to the survival data for patients with IPAH, HPAH and anorexigen associated PAH (23). In contrast, analysis of the US based REVEAL registry showed that patients with PoPH had significantly worse survival when compared to patients with IPAH/FPAH: 67 vs. 85% at 2 years, and 40 vs. 64% at 5 years (Figure 1) (20). These findings were similar in the Spanish REHAP registry, where the 5 year mortality was 49 and 69%, for PoPH and IPAH/HPAH, respectively (Figure 2) (24, 25). Furthermore, severe PoPH is associated with significantly decreased survival in patients undergoing LT (7, 26), and severe PoPH precludes liver transplantation and significantly affects the course of hepatic failure in these patients (26, 27).

The lack of better outcomes in patients with PoPH as compared to their IPAH/FPAH counterparts is not entirely clear, especially in light of their relatively better hemodynamic profile and functional status (20, 25). One possible explanation is increased deaths from liver related events, which account for 25–33% of deaths in patients with PoPH, as compared to only 5% in patients with IPAH (22, 25). In patients with liver disease, higher Child-Pugh Scores are associated with worse outcomes (14, 22), which makes it clear that the severity of liver disease (rather than PoPH) is the main factor in worse outcomes in this population. Another possibility is a relative delay in PAH-specific treatments within the PoPH cohort. PoPH patients started on PAH-specific therapy live longer, despite worse hemodynamics, when compared to PoPH patients not on therapy (25). However, the percentage of PoPH patients on pulmonary vasodilators at both time of enrollment and at 90-days post enrollment into the REVEAL registry was significantly lower than their IPAH/FPAH counterparts (20). The cause of this delay is likely multifactorial, and includes milder symptoms, better hemodynamics, and lack of definitive data to suggest the ideal agent in patients with PoPH.

Likewise, the difference in survival seen between different PoPH cohorts are also likely due to a variety of factors, which include differences in regional screening practices for the presence of PoPH. Patients in the US and Spain with cirrhosis are typically screened at time of liver transplant evaluation, which likely skews the diagnosis of PoPH to patients with more severe liver disease. In contrast, all patients with cirrhosis are screened for PoPH in France, which likely leads to increased rates of diagnosis of PoPH in patients with milder liver disease, which in turn increases the overall survival rates. Rates of referrals of patients to PH specific centers may differ by region (the REVEAL registry included PAH patients treated in the community in addition to academic PH-centers). There may also be differences in baseline characteristics of patients enrolled in each respective registry (including New York Heart Association Functional Class [NYHA FC] and severity of underlying liver disease at the time of enrollment). Additional prospective studies are needed as PoPH-specific management guidelines are established and standardized, to see if this gap can be improved.

## Pathogenesis of PoPH

**Figure 3:**
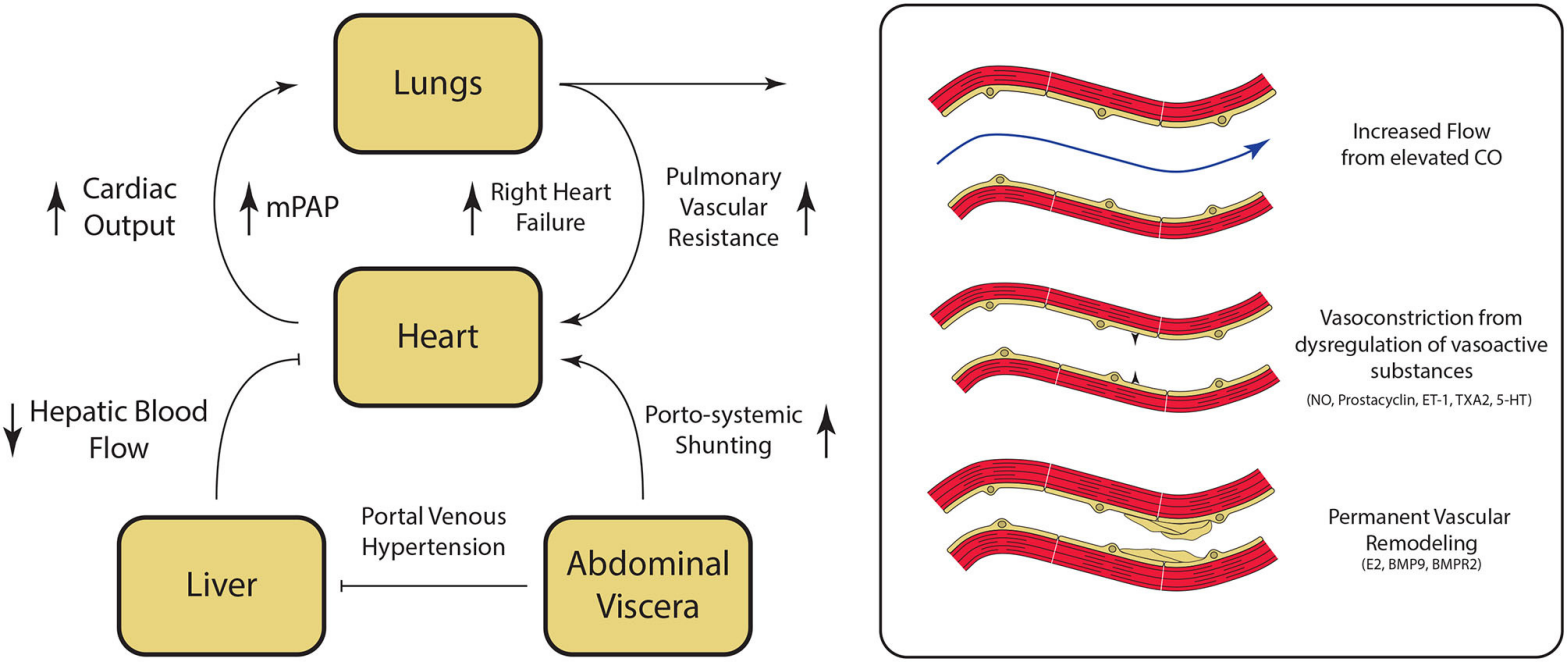
Pathogenesis of Portopulmonary Hypertension (PoPH). Hepatic fibrosis results in portal venous hypertension, increased resistance to hepatic blood flow, and splanchnic vasodilation. Splanchnic vasodilation results in increase in overal circulating volume. and the diversion of blood flow from the liver to the heart via porto-systemic shunting resulting in an overall hyperdynamic state. Dysregulation of key regulator of pulmonary vascular tone associated with cirrhosis results in pulmonary vasoconstriction. At the same time, damage to the pulmonary endothelium and the underlying smooth muscle results in permanent vascular remodeling, which ultimately leads to the development of pulmonary hypertension. CO, cardiac output; NO, nitric oxide; ET-1, endothelin-1; TXA2, thromboxane A2; 5-HT, serotonin; E2, estrogen; BMP9, Bone Morphogenic Protein 9; BMPR2, Bone Morphogenic Receptor 2. Please refer to the text for additional details.

The underlying mechanisms for the development of PoPH are poorly understood and continue to be an area of active research. Liver cirrhosis and portal hypertension lead to splanchnic vasodilatation and formation of portosystemic shunts, which are thought to contribute to PoPH pathogenesis in multiple ways (Figure 3). Mechanically, increased intrahepatic resistance to flow from cirrhosis results in an increased portal pressure gradient and portosystemic collateralization through the reperfusion/dilation of preexisting vessels and through the generation of new vessels (28). On a molecular level, portosystemic shunting enables blood to bypass the liver, and thus evade hepatic metabolism of vasoactive substances. This direct reduction in the peripheral vascular resistance, combined with indirect vasodilation via intestinal vasoactive substances that are now able to bypass the liver and reach systemic circulation, ultimately result in a hyperdynamic state. This phenomenon has been seen in patients immediately after undergoing a transjugular intrahepatic portosystemic shunt (TIPS) placement, and persisted even a month out (29). Likewise, levels of vasoactive substances acting in the pulmonary circulation (including pulmonary vasodilators nitric oxide and prostacyclin, as well as pulmonary vasoconstrictors endothelin-1, thromboxane A2, and serotonin), are similarly dysregulated in cirrhosis (30–32). The imbalance of these vasoactive substances in the pulmonary vasculature results in net vasoconstriction and pulmonary vascular resistance elevation.

Shear stress from persistently high flows leads to endothelial cell injury and the activation and repression of genes that participate in the vascular remodeling process, and is believed to play a role in various forms of PAH (33–35). Exposure of and damage to the underlying arterial smooth muscle results in smooth muscle proliferation and thickening of the tunica intima, media, and adventitia within the pulmonary vasculature (4). The arterial wall thickening, in turn, can result in more sluggish pulmonary blood flow, platelet aggregation, and thrombus formation, which can then become recanalized over time (3). Autopsy data of patients with PoPH confirms that the end stage of this process is medial and intimal thickening, plexiform lesions and fibrotic venular obstruction (36). Together, these vascular lesions result in permanently elevated PVR that distinguishes PoPH from other causes of elevated mPAP in patients with portal hypertension (Figure 5).

Signaling mediated by bone morphogenic protein receptor 2 (BMPR2), a member of the TGF-β superfamily, has been shown to play a key role in familial pulmonary hypertension, and mutations in the gene encoding this receptor have also been found in 15–40% of idiopathic cases of PAH (37, 38). Bone morphogenic protein 9 (BMP9) is a circulating factor produced by hepatic stellate cells, which serves as a ligand for BMPR2 signaling, and has been shown to have a protecting effect against hepatic fibrosis (39). Recent studies have shown that patients with PoPH have significantly lower circulating levels of BMP9 as compared to control patients with advanced liver disease and no evidence of pulmonary hypertension (40). BMP9 levels were likewise significantly lower in patients with PoPH as compared to patients with other etiologies of group 1 PAH (41). Moreover, selective enhancement of endothelial BMPR2 with exogenous BMP9 has been shown to reverse the presence of PAH in multiple mouse models of PAH (42). Together, these findings suggest that BMP9 and BMPR2 associated downstream signaling pathways also likely play a role in the pathogenesis of PoPH.

More recently, activation of toll-like receptors (TLRs) by bacterial lipopolysaccharides (LPS) from bacterial translocation has been implicated in the pathogenesis of PAH. Mouse models deficient in BMPR2 exposed LPS were shown to develop evidence of pulmonary hypertension, which did not occur in their wild-type counterparts (43). The underlying mechanism for this is thought to be rooted in increased IL-6 production and activation of the STAT3 transcription factor, which is associated with increased expression of TLR4 (43). TLR4 is the main receptor for LPS and has been previously show to promote the development of PAH (44–46). Together, these data suggest that exposure to bacterial endotoxins, which occurs more frequently in cirrhotic patients due to recurrent gut bacterial translocations, may provide a “second hit” toward the development of PoPH in an otherwise predisposed host.

Lastly, there is growing evidence that altered sex hormone also contributes to the pathogenesis of PoPH. Estrogen binds to the promoter region of the BMPR2 gene and regulates its expression (47). Polymorphisms in CYP1B1 which metabolizes estrogen were associated with penetrance of PAH in women with BMPR2 mutations, but not men (48). Expression of CYP1B1 has also been shown to be increased in experimental models and human PAH (49). A candidate gene study to identify genetic risk factors in PoPH found single nucleotide polymorphisms (SNPs) in the genes coding for estrogen receptor 1 and aromatase, a critical enzyme in the estrogen metabolism (50). Recently, Al-Naamani et al. showed that in PoPH patients, the risk allele rs7175922 in CYP19A1 was associated with significantly higher levels of estradiol, urinary 2-hydroxyestrogen/16-α-hydroxyestrone (2-OHE/16α-OHE1), plasma levels of dehydroepiandrosterone-sulfate and plasma levels of 16-α-hydroxyestradiol (16α-OHE2) compared to the patients with liver disease but no PoPH (51). 16α-OHE1 leads to the development of PAH in animals via upregulation of miR-29 and has been associated with abnormal markers of insulin resistance (52). 16α-OHE1 has also been shown to increase oxidative-stress related proliferation in pulmonary arterial smooth muscle cells from PAH patients (53). Similarly, Sahay et al. performed qualitative immunohistochemical (IHC) staining of the explanted livers of PoPH patients and compared them with normal and cirrhotic livers. However, limited by the small sample size, they did not find any significant difference between the PoPH and cirrhotic livers, but aromatase expression was increased compared to the normal liver (54). More research is needed to explore the links between altered estrogen metabolism and the development of PoPH in patients with liver disease.

## Screening and Diagnosis of Portopulmonary Hypertension

**Figure 4:**
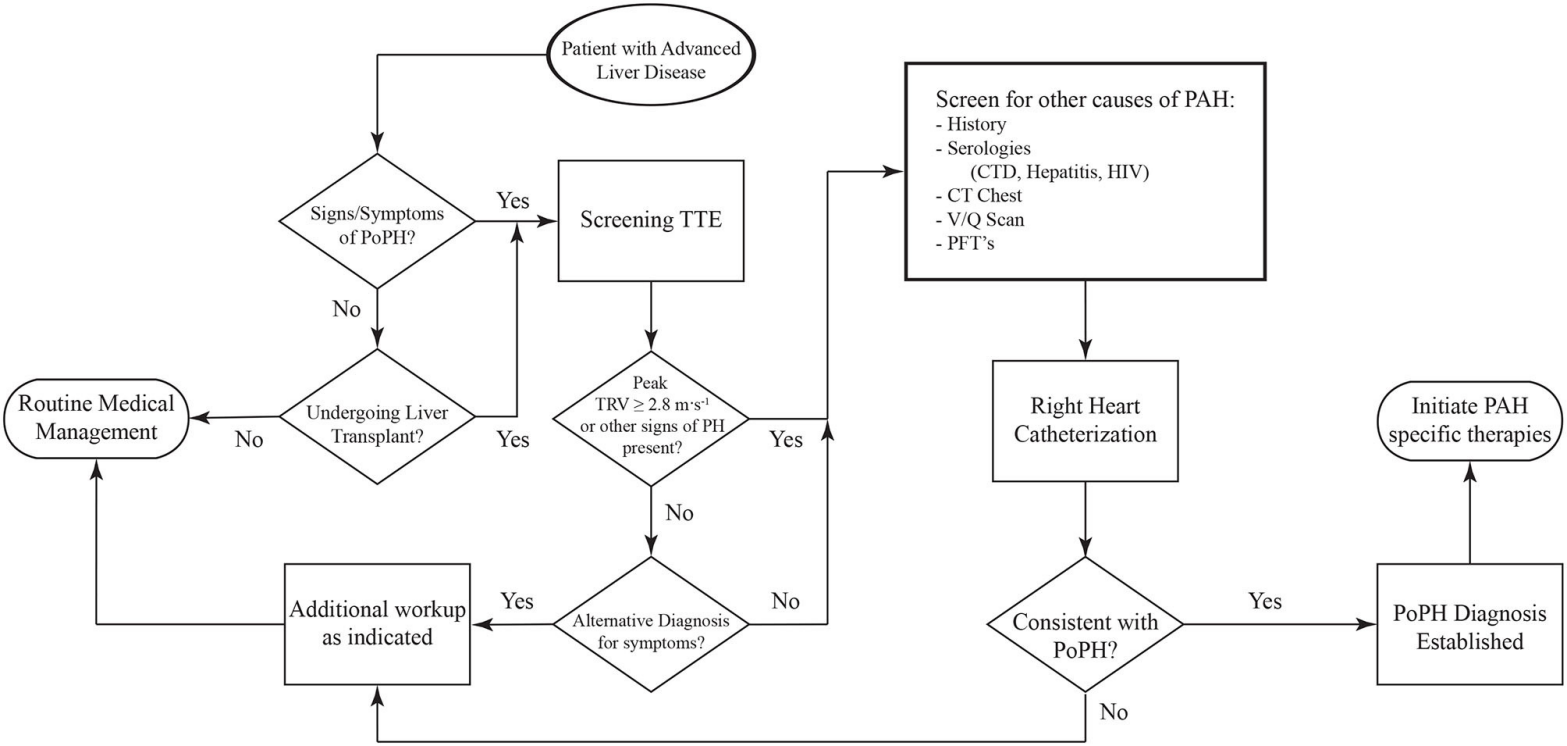
Algorithm for the diagnosis of PoPH. PoPH, Portopulmonary Hypertension; TTE, Transthoracic Echocardiogram; TRV, Tricuspid Regurgitant Velocity; CTD, Connective Tissue Diseases; PFT's, Pulmonary Function Tests.

Screening for PoPH should begin with a detailed history and physical exam (Figure 4). Common symptoms of PoPH include unexplained dyspnea on exertion, fatigue, weakness, orthopnea, near-syncope, syncope, palpitations, and chest pain, with dyspnea being by far the most common. On physical exam, these patients often have an accentuated pulmonic component of the second heart sound (P2), a right sided S3, and a right sided S4. Additionally, the presence of a tricuspid regurgitant murmur or a pulmonic insufficiency murmur can be heard in 40% and 13% of patients, respectively (55). Other signs of right heart failure, including jugular venous distention, ascites, and bilateral lower extremity edema, may also be seen (56). Additional history looking for other known World Health Organization (WHO) Group 1 PAH risk factors should be taken. This includes use of drugs such as methamphetamines, use of dietary supplements, prior history of chemotherapy, and history of autoimmune diseases. Clinicians should also review the medical history for any other conditions known to be associated with PH, such as left heart disease, chronic lung disease, obstructive sleep apnea, venous thromboembolism, and hematologic disorders. If the history and physical arouses any suspicion for PoPH, the patient should undergo an echocardiogram.

Laboratory workup should include serological screening tests for connective tissue diseases, hepatitis and HIV. Additional evaluation with pulmonary function tests (including spirometry, lung volumes, diffusion capacity, and a 6 min walk test) and a CT scan of the chest should be obtained to screen for the presence of significant lung disease, and to establish a baseline which can then be used to track disease progression (11, 57). A ventilation/perfusion (V/Q) scan should also be done to screen for the presence of chronic thromboembolic pulmonary hypertension (CTEPH) (especially in the setting of portal vein thrombosis). See Table 1 for more symptoms, physical exam and imaging findings.

**Table 1:** Comon clinical and diagnostic findings in patients with Pulmonary Hypertension.

**Symptoms**	**Physical exam**	**CT findings**	**TTE findings (57)**
Dyspnea on exertion Fatigue Weakness Orthopnea Near-syncope Syncope Palpitations Chest Pain	Accentuated P2 Right sided S3 Right sided S4 TR Murmur Pulmonic Insufficiency Murmur Jugular Venous Distension Ascites Lower Extremity Edema Cyanosis	PA diameter ≥29 mm PA to Ascending Aorta diameter ratio of ≥1 RA Dilation RV Dilation	Peak TR velocity > 2.8 m/s RV/LV basal diameter > 1.0 Flattening of interventricular septum RV outflow Doppler acceleration <105 msec Early diastolic pulmonary regurgitation velocity >2.2 m/s PA diameter > 25 mm IVC diameter >21 mm RA end-systole area >18 cm^2^

*TR, Tricuspid Regurgitation; PA, Pulmonary Artery; RA, Right Atrium; RV, Right Ventricle; LV, Left Ventricle; IVC, inferior vena cava*.

Transthoracic echocardiography (TTE) is the best screening test for PoPH, as it is for other forms of PAH. All patients with portal hypertension should be screened for PoPH with a TTE. A list of the most common echocardiographic findings that are seen in patients with pulmonary hypertension can be seen in Table 1. Although traditionally used throughout the literature to screen for the presence of pulmonary hypertension, right ventricular systolic pressure (RVSP) calculations using the simplified Bernoulli equation are prone to error, stemming from the difficulty in accurately assessing a patient's right atrial pressure. In their analysis of echocardiographic screen of patients undergoing liver transplant evaluation, Krowka et al. demonstrated only moderate correlation between the RVSP and the PA systolic pressures that were measured on right heart catheterization (RHC) (19). Moreover, this discrepancy increased with increasing values of RVSP. Likewise, cutoff values of RVSP used for ruling out the presence of PoPH have differed between studies, and the ideal threshold value for a positive screen has been a matter of debate. As such, it has been suggested that the use of RVSP should be replaced by continuous wave Doppler measurements of peak tricuspid regurgitant velocity, with cutoff values of 2.8 m·s^−1^ for intermediate and 3.4 m·s^−1^ for high probability for the presence of PH (58). Issues with calculating RVSP notwithstanding, current guidelines from the American Association for the Study of Liver Diseases (AASLD) recommend evaluation with right heart catheterization in all patients being considered for LT with an RVSP ≥45 mmHg on screening echocardiogram (59). The International Liver Transplant Society (ILTS) guidelines recommend a RHC in patients with RVSP > 50 mmHg and/or evidence of right ventricular (RV) hypertrophy or dysfunction on TTE (60). Clinicians should understand that when lower RVSP cutoffs are used, the sensitivity will approach 100%, but the specificity will decrease, resulting in a significant number of false positives (17). However, given the high mortality associated with severe PoPH and LT, performing an unnecessary RHC is preferable to “missing” PoPH. In practice, all patients in whom there are TTE findings suspicious for PoPH (elevated RVSP or signs of RV dysfunction) should undergo RHC.

## Right Heart Catheterization Hemodynamics Interpretation in PoPH

The diagnosis of PoPH includes hemodynamic evaluation of both the pulmonary and hepatic circulations. Portal hypertension can be challenging to diagnose non-invasively. Esophageal varices and skin collaterals are suggestive of portal hypertension. On ultrasound, decreased portal vein flow velocity, and portal vein biphasic or flow reversal are suggestive of portal hypertension (61). A hepatic venous pressure gradient (HVPG) (wedge hepatic vein pressure [WHVP]—free hepatic vein pressure [FHVP]) of >5 mmHg is diagnostic of sinusoidal portal hypertension (62).

On RHC, the patient must have both an elevated mPAP and PVR and a normal pulmonary artery wedge pressure (PAWP) for a positive diagnosis of PoPH. During the most recent World Symposium on Pulmonary Hypertension, the hemodynamic criteria for the diagnosis of pre-capillary pulmonary hypertension (which includes WHO Group 1) were revised. With this change, patients with a mPAP > 20 mmHg (previously > 25 mmHg), a PVR of ≥ 3 Wood units (WU) (≥240 dynes), and a PAWP < 15 mmHg meet the diagnostic criteria for the presence of PAH (1).

**Figure 5:**
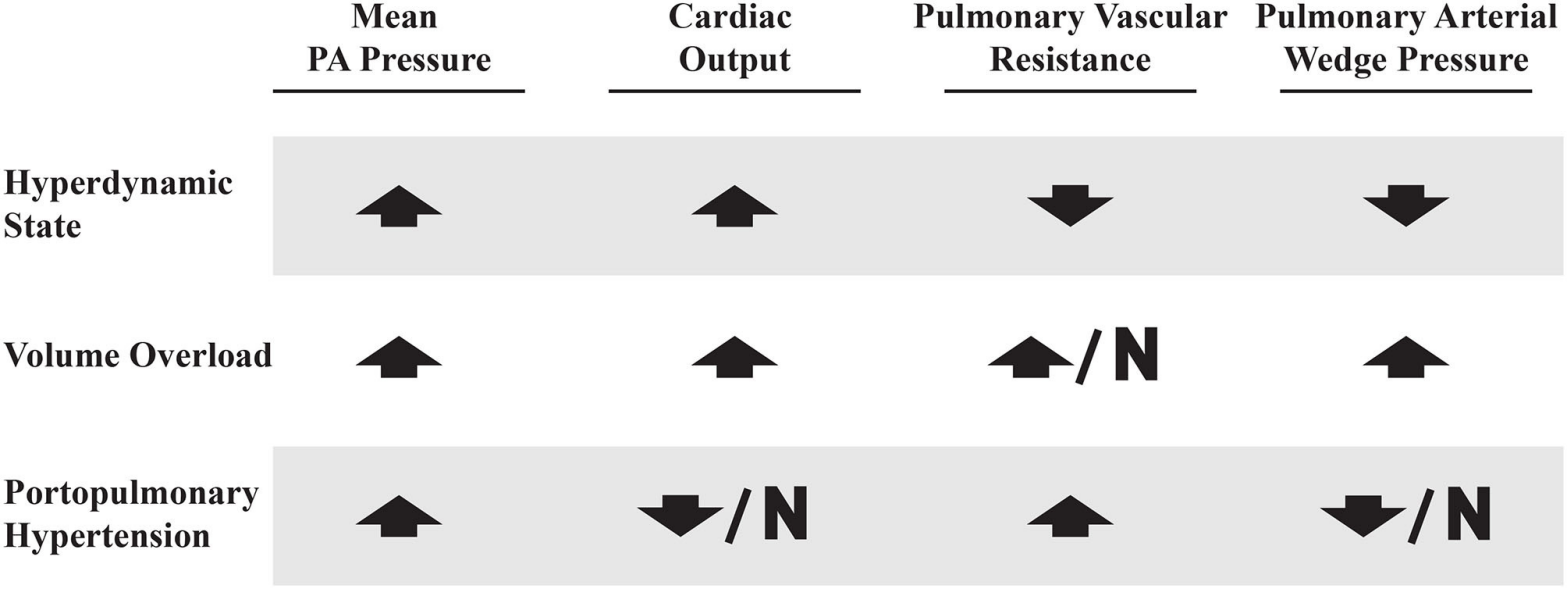
Right heart catheterization profiles in patients with elevated mean PA pressures. Up arrow, increased; Down arrow, decreased; N, normal.

Careful evaluation of a patient's hemodynamic profile on RHC is crucial in order to distinguish PoPH from other clinical conditions that are common in patients with liver disease that can also result in elevated mPAPs. These conditions can be classified into three broad categories: hyperdynamic state, volume overload and true portopulmonary hypertension (63). See Figure 5 for comparisons between these hemodynamic profiles.

Patients with decompensated liver disease may present with an elevated mPAP, PAWP, and PVR. This may be due to a combination of volume overload, hyperdynamic state, and PoPH (perhaps all three). Volume overload is managed with careful diuresis, as patients with liver disease are prone to kidney injury (64). A hyperdynamic state can be intrinsic to liver disease, but may also be due to other treatable medical comorbidities (such as anemia, arteriovenous shunts, and hyperthyroidism) (65–67). This common clinical scenario speaks to the challenge of diagnosing and treating PoPH, which is best done at expert centers which are most familiar with these complicated patients.

## Treatment of PoPH

The importance of screening for PoPH was established by Krowka et al. in a retrospective review of case reports and case series, which looked at the relationship between the presence of untreated PoPH and cardiopulmonary related mortality in patients undergoing LT (7). In this seminal work, Krowka et al. examined 43 patients with PoPH (confirmed by RHC) who were not on medical treatment and underwent LT. The patients were grouped based on severity of pulmonary hypertension. Patients with a mPAP between 25 and 35 mmHg were considered to have mild disease, those with a mPAP between 35 and 50 mmHg were considered moderate, and those with a mPAP >50 mmHg were considered severe. In these groups, 100% of the patients with severe and 50% of the patients with moderate PoPH died due to cardiopulmonary related events (the majority died during the transplantation hospitalization). No mortality was reported among the patients with mild PoPH (mPAP < 35 mmHg). As a result of this study, and others, routine screening for the presence of pulmonary hypertension is now recommended in all patients undergoing evaluation for LT.

After this landmark study established that PoPH patients with higher mPAP are at high risk of mortality with LT, it prompted the question of whether medical therapy could mitigate some of those risks. Treatment of PoPH prior to or instead of liver transplantation remains challenging and is fraught with a paucity of data and large knowledge gaps. Randomized controlled trials of pulmonary hypertension specific medical therapies for patients with portopulmonary hypertension have been limited by concerns regarding adverse hepatic effects of PAH specific medications (68). We will review select clinical trials of PAH-specific therapy in PoPH, as well as the indications and contraindications for liver transplantation.

Retrospective data on treatment of PoPH is quite heterogenous, with some studies showing improvement in hemodynamics and outcomes, and others showing no treatment effect. There is higher quality data from prospective non-randomized studies, from which a few themes emerge. A recently published prospective cohort study from the French Pulmonary Hypertension Registry (FPHR) examined data on 637 patients with PoPH, 90% of whom were started on PAH-specific therapies (12). These patients experienced significant improved WHO functional class, 6-min walk distance (6MWD) and hemodynamic parameters. The ANGEL study is a recent single arm prospective trial of ambrisentan in 31 patients with PoPH (69). This trial showed improved hemodynamics (PVR, mPAP, cardiac index [CI]) and functional class, but no change in 6MWD. From these prospective studies, it is reasonable to conclude that PAH directed therapy has favorable effects on hemodynamics and functional outcomes, but the effects on mortality and candidacy for LT are unclear.

As previously stated, the vast majority of randomized controlled trials of PAH-specific therapies in PAH patients have excluded patients with PoPH. One notable exception is the PATENT-1 trial by Ghofrani et al. (phase 3, double-blind, randomized controlled trial), which randomized 443 patients with various etiologies of WHO group 1 PAH to receive either placebo or two different doses of riociguat (70). This trial is significant in that it included 13 patients with PoPH, 11 of whom received the highest dose of study medication (2.5 mg daily). The primary end point of the PATENT-1 trial was 6MWD, which was significantly improved in the riociguat 2.5 mg group. Secondary hemodynamic (PVR, mPAP, and CO) and functional (WHO functional class, Borg dyspnea scale and time to clinical worsening) endpoints were also significantly improved in the riociguat 2.5 mg daily group. The PATENT-2 trial was an extension of the PATENT-1 study, in which all eligible patients from the first study were given riociguat and followed for 2 years. PATENT-2 showed sustained improvements in functional class and 6MWD (71). A recently published subgroup analysis of the 13 patients with PoPH who were enrolled in PATENT-1 and 2 showed similar improvements in WHO functional class, 6MWD and PVR (72). Importantly, riociguat was well-tolerated by the PoPH patients.

The first ever randomized controlled trial of PAH specific therapy in patients with PoPH was published in 2019. The PORTICO study, which randomized 85 patients with PoPH to receive either macitentan or placebo for 12 weeks, found that the macitentan group had a 35% reduction in PVR, the primary endpoint (73). While there were significant improvements in some secondary endpoints (mPAP and CI), there were no significant improvements in WHO functional class and 6MWD. Importantly, macitentan was well-tolerated in this population, with no hepatic side effects.

A notable limitation of PORTICO is that it excluded patients with Child-Pugh class C liver disease and patients with a MELD score of >19, which are groups with known poor survival (12).

It is still unclear whether this significant reduction in PVR could translate into more meaningful outcomes for patients (improved functional capacity, decreased mortality) or alter candidacy for liver transplant. However, a recently published *post-hoc* analysis of the PORTICO study found that patients in the macitentan treatment group had significantly decreased waitlist and perioperative mortality risk categories as compared with the placebo arm (74).

Taken together, these studies show that PAH specific therapies can improve hemodynamics and functional outcomes in patients with PoPH. Importantly, these medications seem to be well-tolerated in patients with liver disease and PoPH. By extension, they can help patients become candidates for LT and potentially live longer. In the absence of PoPH specific treatment guidelines, clinicians should follow the general treatment principles of PAH, with special attention to the unique considerations for patients with PoPH. More research is needed to elucidate the nuances of PAH specific therapies in the PoPH population.

## Liver Transplantation

As previously discussed, the severity of PoPH is an established risk factor for liver transplant waitlist mortality (75) and poor perioperative outcomes (7). As such, decisions about candidacy for liver transplantation in patients with PoPH are complex and best done by experienced centers.

The following general principles are recommended by the AASLD (59) for management of patients with liver disease who are being considered for LT. All patients who are undergoing evaluation for LT should be screened for PoPH with transthoracic echocardiography, and patients with RVSP > 45 mmHg should be further evaluated by RHC. Patients with mPAP < 35 mmHg do not require treatment with PAH specific therapies and can proceed with further evaluation for LT. Patients with mPAP > 35 mmHg should be evaluated by an experienced PH specialist and be considered for PAH specific therapy. If the mPAP can be lowered to <35 mmHg and the PVR can be lowered to <5 WU with medical therapy, LT can be pursued.

For some patients with mild liver disease and PoPH, their higher risk for waitlist mortality is not accurately captured by the MELD score (76). LT candidates with PoPH are eligible for MELD exception points if the following criteria are met: initial mPAP > 35 and PVR > 3 WU; patients are treated with PAH specific therapy; and post-treatment mPAP is lowered to <35 mmHg (76).

Giving MELD exception points to patients with PoPH is controversial for a number of reasons. The U.S. based Organ Procurement and Transplant Network (OPTN) requires a RHC and resubmission of full hemodynamic data every 3 months (76) to maintain the MELD exception points, which is burdensome and often impractical for both patients and institutions. Despite having to submit full hemodynamic data every 3 months, only mPAP < 35 mmHg is used to maintain eligibility MELD exception points. Some patients may achieve a lower PVR with PAH directed therapy, but continue to have an elevated mPAP from volume overload and/or a high flow state (60). In an analysis of patients with PoPH on the LT waitlist, initial PVR was an independent predictor of waitlist mortality, while pre- and post-treatment mPAP were not (75). This suggests that a broader hemodynamic picture should be used to maintain MELD exception points eligibility.

In one analysis of 155 patients on the LT waitlist with approved PoPH MELD exception points, less than half of the patients actually met the standardized OPTN MELD exception criteria. Furthermore, almost one third of patients in this study lacked sufficient hemodynamic data to diagnose PoPH or had hemodynamic data that was inconsistent with PoPH (77). These researchers found that overall mortality was higher for patients given PoPH MELD exception points (as compared to patients listed without exception points), which shows the need for more research into methods of risk stratification for patients with PoPH.

In the era of the AASLD/ILTS guidelines and MELD exception points, data on the natural course of PoPH after LT is limited. In one retrospective cohort study, hemodynamics and survival data for 35 patients with PoPH were examined (78). After LT, 6 month, 1 year, and 3 year survival rates were 80, 77, and 77%, respectively. Of the 27/35 patients who survived more than 6 months after LT, all were able to be weaned from intravenous epoprostenol. Furthermore, most of the patients had improvement in their hemodynamics after LT, with 30% having a mPAP < 25 mmHg at last follow up. This data suggests that carefully selected patients with PoPH who undergo LT will have significant improvement in their hemodynamics.

## Challenges in the Diagnosis and Management of PoPH

There are a number of barriers to the effective diagnosis and treatment of patients with PoPH. As previously discussed, accurate diagnosis of PoPH is challenging, and the differential for an elevated mPAP in the setting of liver disease is broad. There is only one randomized controlled trial for PAH specific therapy in patients with PoPH. Advanced liver disease symptoms have significant overlap with the side effects of PAH specific therapies (e.g., nausea, fatigue, and fluid overload), which can make the initiation and maintenance of treatment challenging (67).

Many of the drugs used to treat PoPH are metabolized in the liver, and drug metabolism is altered by liver disease and other medications that patients may be taking (67). Additionally, patients may have hepatic encephalopathy or lack the social support needed to safely administer parenteral therapy or take medications multiple times per day (67). More generally, many of the treatment barriers that apply to all patients with PAH apply to patients with PoPH: late referrals, lack of an expert center in close proximity to where the patient lives, and insurance issues. There is significant variability in the screening and management practices of PoPH, discordance between published guidelines and actual practice patterns, and disagreements about the role of LT in PoPH (79).

## Conclusions and Areas of Future Focus

PoPH remains a challenging disease entity with many facets in need of further research. More research is needed into which types of PAH specific therapies will benefit patients with varying severities of PoPH. The first randomized control trial in PoPH was recently published, which showed that macitentan significantly lowered PVR in patients with PoPH. It unfortunately remains unclear if hemodynamics improvements translate into successful LT or substantial survival benefit. While it is clear that patients with severe disease have high mortality with or without LT, their long-term response to treatment is unknown. It is unknown if mild PoPH needs to be treated or given MELD exception points, and the risk for progression to more severe disease is unknown.

Pre-operative risk stratification of patients prior to LT remains a challenge, as the traditional cut off of a mPAP of 35 mmHg is somewhat arbitrary and is based on a small retrospective review of case reports and series. There are no published guidelines on initiation of PAH specific therapies in PoPH, or whether to continue PoPH treatment post-LT. Finally, there is no centralized national registry of PoPH patients who undergo LT; this would serve as a foundation for focusing on the aforementioned areas of need.

While there are indeed many barriers to and challenges in caring for patients with PoPH, it remains a field with many areas of active investigation. As the mechanisms and treatment continue to be explored, we are confident that care for these complex patients will continue to improve.

## Author Contributions

CT and VG: primarily responsible for writing the manuscript. VJ and SS: editing and revision and mentoring. All authors contributed to the article and approved the submitted version.

## Conflict of Interest

SS is a speaker and consultant for J&J, Bayer, United Therapeutics, Advisor for Liquidia, and Gossamer biotech. SS has received prior and current research grants from ACCP CHEST, and is a clinical trial end point adjudication committee member in a trial sponsored by GSK. The remaining authors declare that the research was conducted in the absence of any commercial or financial relationships that could be construed as a potential conflict of interest.
